# Key co-expressed genes correlated with blood serum parameters of pigs fed with different fatty acid profile diets

**DOI:** 10.3389/fgene.2024.1394971

**Published:** 2024-07-03

**Authors:** Simara Larissa Fanalli, Júlia Dezen Gomes, Francisco José de Novais, Izally Carvalho Gervásio, Heidge Fukumasu, Gabriel Costa Monteiro Moreira, Luiz Lehmann Coutinho, James Koltes, Andreia J. Amaral, Aline Silva Mello Cesar

**Affiliations:** ^1^ Faculty of Animal Science and Food Engineering, (FZEA), University of São Paulo, SãoPaulo, Brazil; ^2^ Department of Animal Science, Luiz de Queiroz College of Agriculture, University of São Paulo (USP), Piracicaba, Brazil; ^3^ Department of Agricultural, Food & Nutritional Science, Faculty of Agricultural, Life and Environmental Science, University of Alberta, Edmonton, AB, Canada; ^4^ INRAE, AgroParisTech, BREED, Université Paris Saclay, Jouy-en-Josas, France; ^5^ Animal Science Department, Iowa State University, Ames, IA, United States; ^6^ Mediterranean Institute for Agriculture, Environment and Development (MED), Évora, Portugal; ^7^ Centre for Interdisciplinary Research in Animal Health (CIISA), Faculty of Veterinarian Medicine, University of Lisbon, Lisbon, Portugal; ^8^ Department of Food Science and Technology, Luiz de Queiroz College of Agriculture, University of São Paulo (USP), Piracicaba, Brazil

**Keywords:** lipid metabolism, WGCNA, co-expression, RNA-seq, transcriptome, soybean oil, immune response, systems biology

## Abstract

This study investigated how gene expression is affected by dietary fatty acids (FA) by using pigs as a reliable model for studying human diseases that involve lipid metabolism. This includes changes in FA composition in the liver, blood serum parameters and overall metabolic pathways. RNA-Seq data from 32 pigs were analyzed using Weighted Gene Co-expression Network Analysis (WGCNA). Our aim was to identify changes in blood serum parameters and gene expression between diets containing 3% soybean oil (SOY3.0) and a standard pig production diet containing 1.5% soybean oil (SOY1.5). Significantly, both the SOY1.5 and SOY3.0 groups showed significant modules, with a higher number of co-expressed modules identified in the SOY3.0 group. Correlated modules and specific features were identified, including enriched terms and pathways such as the histone acetyltransferase complex, type I diabetes mellitus pathway, cholesterol metabolism, and metabolic pathways in SOY1.5, and pathways related to neurodegeneration and Alzheimer’s disease in SOY3.0. The variation in co-expression observed for HDL in the groups analyzed suggests different regulatory patterns in response to the higher concentration of soybean oil. Key genes co-expressed with metabolic processes indicative of diseases such as Alzheimer’s was also identified, as well as genes related to lipid transport and energy metabolism, including *CCL5, PNISR, DEGS1.* These findings are important for understanding the genetic and metabolic responses to dietary variation and contribute to the development of more precise nutritional strategies.

## 1 Introduction

Fatty acids (FA) have an important role in controlling gene expression and regulating various signaling pathways by binding to specific transcription factors (Larsen et al., 2018). The ability to influence gene transcription impacts lipid metabolism, such as polyunsaturated fatty acids (PUFA) modulating low-density lipoprotein (LDL) and other genes related to lipid metabolism (Denke, 2006; Larsen et al., 2018). According to Larsen et al. (2018), a dietary FA alters plasma lipids, thereby affecting the risk of cardiovascular disease (CVD). Therefore, maintaining cholesterol balance is vital for a healthy life, including a healthy liver, which plays an important role in lipid metabolism and balance (Cohen, 2008).

The liver is responsible for eliminating excess cholesterol that is carried on lipoprotein particles such as high-density lipoprotein (HDL), intermediate-density lipoprotein (IDL), LDL, and remnants of chylomicrons (Maxfield and Tabas, 2005). In addition, very low-density lipoprotein (VLDL) is composed primarly of triglycerides that are assembled in the liver and then released into the bloodstream (Wondmkun, 2020; Zhou and Sun, 2021). Moreover, in lipid metabolism, compounds such as albumin, a predominant protein in mammalian blood plasma, function as carriers of compounds, including FA, facilitating their transport into cells (Vusse, 2009; Junk et al., 2010).

Pork consumption has economic importance and nutritional value for cultural consumers, including the FA profile, which has a direct impact on human health (OECD-FAO, 2021; Fanalli et al., 2022a). The consumption of unsaturated fatty acids, such as linoleic acid (LA, C18:2 cis 9, 12) and oleic acid (OA, C18:1 cis 9) has been related with health benefits, such as reduction of total and LDL cholesterol (Lunn and Theobald, 2006) and beneficial in anti-inflammatory and vascular activities (Sakurai et al., 2021), respectively. Furthermore, feeding pigs diets that are rich in vegetable oils containing unsaturated FA can lead to the production of healthier meat products, depending on changes in the animal’s lipid profile (Alencar et al., 2021; Fanalli et al., 2022c)

The chemical composition of blood serum in pigs reflects the health, nutritional status, and breeding conditions to which they have been exposed. Plasma protein levels have already been used to study the genetic control of disease resilience in pigs (Chen et al., 2023). As a result, the molecular contributions of variation on blood calcium (Ca) and phosphorus (P) levels and identifying putative genes and associated QTL regions (Reyer et al., 2019) and serum parameters for predict total Ca, P intake and identify biomarkers (Vötterl et al., 2021). However, studies on the biochemical parameters in pigs remain limited. Pigs are widely acknowledged as a reliable model for studying human diseases that involve lipid metabolism, such as diabetes, metabolic syndrome, obesity, and cardiovascular disease (Miller and Ullrey, 1987; Zhang et al., 2020; Pan et al., 2021; da Silva et al., 2023).

In this context, identifying gene networks associated with biological pathways helps us understand the mechanism of gene regulation in the liver of pigs fed diets with different levels of soybean oil. The Weighted Correlation Network Analysis (WGCNA) is widely used and has a crucial aspect of systems biology approaches (Kogelman et al., 2014). In our previous study, the FA profile influenced gene expression, resulting in the identification of 281 differentially expressed genes (DEG) among animals that consumed soybean oil at different levels (1.5% and 3%). This changed the composition of FA deposited in the liver, blood parameters, and metabolic pathways, as well as the network of processes in the liver of the animals (Fanalli et al., 2022c). Therefore, the aim of this study was to further investigate and analyze the relationships between the expressed genes and blood serum parameters. We investigated the systemic biological effects of different levels of soybean oil (1.5% and 3%) added to the diet using the WGCNA method.

## 2 Methods

### Ethics statement

The experimental procedures involving animals are in accordance with the requirements of the Animal Care and Use Committee of Luiz de Queiroz College of Agriculture’s requirements (University of São Paulo, Piracicaba, Brazil; protocol: 2018.5.1787.11.6 and CEUA number: 2018–28) and are conducted in accordance with Guide for the Care and Use of Agricultural Animals in Agricultural Research and Teaching (Fass, 2010).

### 2.1 Animal, and experimental diets

In this study, we used 32 immunocastrated male Large White breed pigs that were homozygous negative for the halothane gene (NN). Animals used in the study were genotyped for the malignant hypothermia mutation in the RYR1 gene (Fujii et al., 1991). The feeding trial included the growth and finishing phases (98 days); for more information on the animals, diet and experimental design, can be found in from Almeida et al. (2021); Fanalli et al. (2022b).

Throughout the experimental period, all pigs had *ad libitum* access to food and water. The experimental diets comprised formulations based on corn and soybean meal. The experimental diets consisted of corn–soybean meal growing–finishing diets, supplemented with either 1.5% soybean oil (SOY1.5) or 3% soybean oil (SOY3.0) These formulations were adjusted based on the growth and finishing phases, with the percentages representing the proportion of soybean oil in relation to the total diet formulation. The diets were formulated to meet or exceed requirements (Rostagno, 2011). In the SOY1.5 group, 16 animals received a diet containing 1.5% soybean oil, and in the SOY3.0 group, 16 animals received a diet containing 1.5% soybean oil (Supplementary Table S1).

### 2.2 Blood biochemical parameters

Phenotypic information included serum parameters obtained from animal blood. Blood samples were collected from the jugular vein 4 days before slaughter and immediately transferred to non-anticoagulant vacuum tubes (Becton Dickinson Vacutainer Systems, Franklin Lakes, NJ, United States) as described previously (Fanalli et al., 2022b). The parameters used for analysis were aspartate aminotransferase (AST), albumin, glucose, total protein, triglycerides, globulin, cholesterol, low-density lipoprotein (LDL), high-density lipoprotein (HDL), and very low-density lipoprotein (VLDL).

Analyzes included serum lipids and biochemistry were analyzed by the Mindray, BS120 (Guangdong, China). Quantification of total cholesterol and fractions was also performed by the enzymatic-colorimetric method, but by selective precipitation. This procedure was carried out using commercial kits following the manufacturer’s instructions. Blood serum glucose was quantified by the colorimetric enzymatic method according to Trinder (Trinder, 1969), using commercial kits according to the manufacturer’s recommended use. The analysis for the determination of total proteins was performed with commercial kits, following the protocol suggested by the manufacturer, using the Biureto method with some modifications (Fanalli et al., 2022b).

### 2.3 RNA-Seq and data analysis

Briefly, total RNA was extracted from the right hepatic lobe. All samples were then sequenced using the TruSeq PE Cluster Kit v4-cBot-HS kit (Illumina, San Diego, CA, United States). Clustering and sequencing were performed on the HiSeq 2500 instrument (Illumina, San Diego, CA, United States) using a TruSeq SBS Kit v4-HS (200 cycles) according to the manufacturer’s instructions (Fanalli et al., 2023).

The sample accession of the mRNA expression data of the PRJEB50513 [http://www.ebi.ac.uk/ena/data/view/PRJEB50513].

The RNA-Seq data quality control was checked using the FastQC, v.0.11.9 [https://www.bioinformatics.babraham.ac.uk/projects/fastqc]. To remove adapters and bases with low PHRED scores we use Trim Galore v. 0.6.5. The tagging of duplicate reads was performed using the Picard Mark Duplicates v. 1.8.x [https://broadinstitute.github.io/picard/]. The initial ten nucleotides of each read were removed.

Quality control and reads statistics were estimated using the RNA-Seq data analysis pipeline used by the EURO-FAANG group within the scope of the H2020 projects, namely, the BovReg project. The entire analysis of workflow was performed by a Nextflow manager (DI Tommaso et al., 2017). Briefly, after selecting high quality reads, they were mapped to the reference genome *Sus scrofa* 11.1 [http://www.ensembl.org/Sus_scrofa/Info/Index] using the Bowtie2 v.2.4.3 and RNA-Seq by Expectation Maximization (RSEM) v. 1.3.1 were used for the estimation of expression values (Li and Dewey, 2011).

### 2.4 Network analysis with weighted correlation network analysis (WGCNA)

The phenotypic data were centered and scaled (Li et al., 2018; Diniz et al., 2019), then a linear model was fitted in R (R Core Team, 2023), prior to analyze co-expression. An adjustment was performed using the equation: $y^{*}=\mu +y-X\hat{\beta \;}+\varepsilon$ , where $y^{*}$ represents the adjusted/corrected phenotype, μ is the general mean vector for phenotypes, y is the vector of phenotypes, X is the incidence matrix for fixed effects, $\hat{\beta }$ is the vector of fixed effects, and ε is the residual vector. Fixed effects including block and sire were used, therefore the block was defined by the weight at the time of exit from the nursery phase and at the time of entry into the experiment in the growth phase.

Parallel, gene co-expression networks were generated by using the WGCNA package in R (Langfelder and Horvath, 2008), with RNA-Seq data. The gene abundance was normalized by transcript per million (TPM) counts, to perform comparison among the samples using RSEM v.1.3.1 (Li and Dewey, 2011; Zhao et al., 2021).

The removed genes included unexpressed genes, defined as those with zero counts across all samples, and infrequently expressed genes, which were genes do not present in at least 50% of the samples.

The similarity among the gene expression profiles of samples in each of the diets was assessed by creating a similarity matrix through the calculation of Pearson’s correlations. Subsequently, the similarity matrix was converted into an adjacency matrix (A) using a β exponent, adhering to the free-scale topology (*R* ^2^ > 0.80) (Diniz et al., 2019; Silva-Vignato et al., 2019). We chose to use signed networks because they are better suited for capturing gene expression trends, including up and downregulation, and for classifying co-expressed gene modules (Langfelder and Horvath, 2008). To define the topological overlap matrix (TOM) modules based on dissimilarity (1-TOM) were used (Langfelder and Horvath, 2008). Modules were merged based on the dissimilarity between the eigengenes. We computed both the correlation matrix and the adjacency matrix, subsequently merging them into the topology matrix. Subsequently, we identified gene modules characterized by dissimilarities less than 0.25, equivalent to a correlation of 0.75, with a minimum module size set at 30 genes. Genes without clusters were grouped in the *Grey* module (Silva-Vignato et al., 2019).

Module-trait associations were assessed through the correlation between the module eigengene (ME) for each blood serum parameter, enabling the identification of modules correlated with specific traits. Gene significance (GS), which refers to the correlation between a gene and a trait, and module membership (MM), which refers to the correlation between an individual gene and the module eigengene, were calculated to validate the module-trait correlation (Zhang and Horvath, 2005; Zhang et al., 2022). For functional enrichment analysis, module genes were selected that showed significant associations (*p*-value <0.05) with at least one trait.

### 2.5 Functional enrichment analysis of co-expressed modules

Functional enrichment analysis was performed using the DAVID database (Sherman et al., 2022) (v. 2021). All nodes were considered after applying the weight threshold, with ME being significantly correlated with fatty acids using a significance criterion of *p*-value <0.05. The cut-off criterion for Gene Ontology (GO) terms (Ashburner et al., 2000) and Kyoto Encyclopedia of Genes and Genomes (KEGG) pathways (Kanehisa and Goto, 2000; Kanehisa, 2019) was set at *p*-value <0.05. We selected the terms GO and KEGG pathway based on their relationship to metabolism, immunity, disease and FA.

### 2.6 Mining and detection of hub genes of co-expression modules

The weight threshold filter was used to retain higher weight connections. The weight assigned to each interaction reflects the degree of biological similarity between two proteins, ranging from 0 to 1. To maintain robustness, proteins with very low functional similarity were excluded from the analysis (Karaca, et al., 2024)

In all constructed networks, our goal was to use the most accurate edge quantification possible, while maintaining a threshold that minimizes disruption to the network, following Zhang’s recommendations (Zhang and Horvath, 2005).

Additionally, the stringApp plugin with STRINGFy function—STRING (Search Tool for the Retrieval of Interacting Genes/Proteins) app was used.

We identified the hub gene using on the following criteria: 1) MM vs. GS with a correlation greater than 0.20 and a *p-*value of <0.05; 2) we visualized the most significantly correlated genes with a WGCNA edge weight using Cytoscape (Shannon et al., 2003), utilizing the edges provided by the WGCNA “exportNetworkToCytoscape” function; 3) we applied the MCODE clustering algorithm (Wang et al., 2015) to identify densely connected subnetworks or modules. Default parameter settings, including “DegreeThreshold,” “NodeScoreThreshold,” “K-CoreThreshold” and “MaxDepth,” were used to extract a highly connected core subnetwork; 4) we obtained annotation information using the STRINGFy network function of the STRING app; 5) as a final step to identify the hub gene, we used the Maximal Clique Centrality (MCC) algorithm available in CytoHubba. This algorithm is a measure of the importance of nodes within a biological network (Chin et al., 2014).

## 3 Results

### 3.1 Construction and analysis of co-expression modules

We used RNA-Seq data obtained from liver samples from pigs fed diets containing two different levels of soybean oil. These data were used to construct a gene expression matrix consisting of 15,912 genes from 17 samples for the SOY1.5 group. During hierarchical clustering of the SOY1.5 samples, sample L7 was identified as an outlier and subsequently removed from the analysis, leaving a total of 16 samples. For the SOY3.0 group, we used 16 samples, as determined by statistical analysis in previous work, and generated a gene expression matrix comprising 16,098 genes (Fanalli et al., 2022b). Signed gene co-expression networks (i.e., modules) were identified with the WGCNA package in R. Supplementary Table S2 shows animal identification, and phenotypic values (blood serum parameters). The same sequential input relationship was followed for blood serum parameters. Following the scale-free network criteria, we select an appropriate weighting parameter for the adjacency function (Zhao et al., 2022).

**FIGURE 1:**
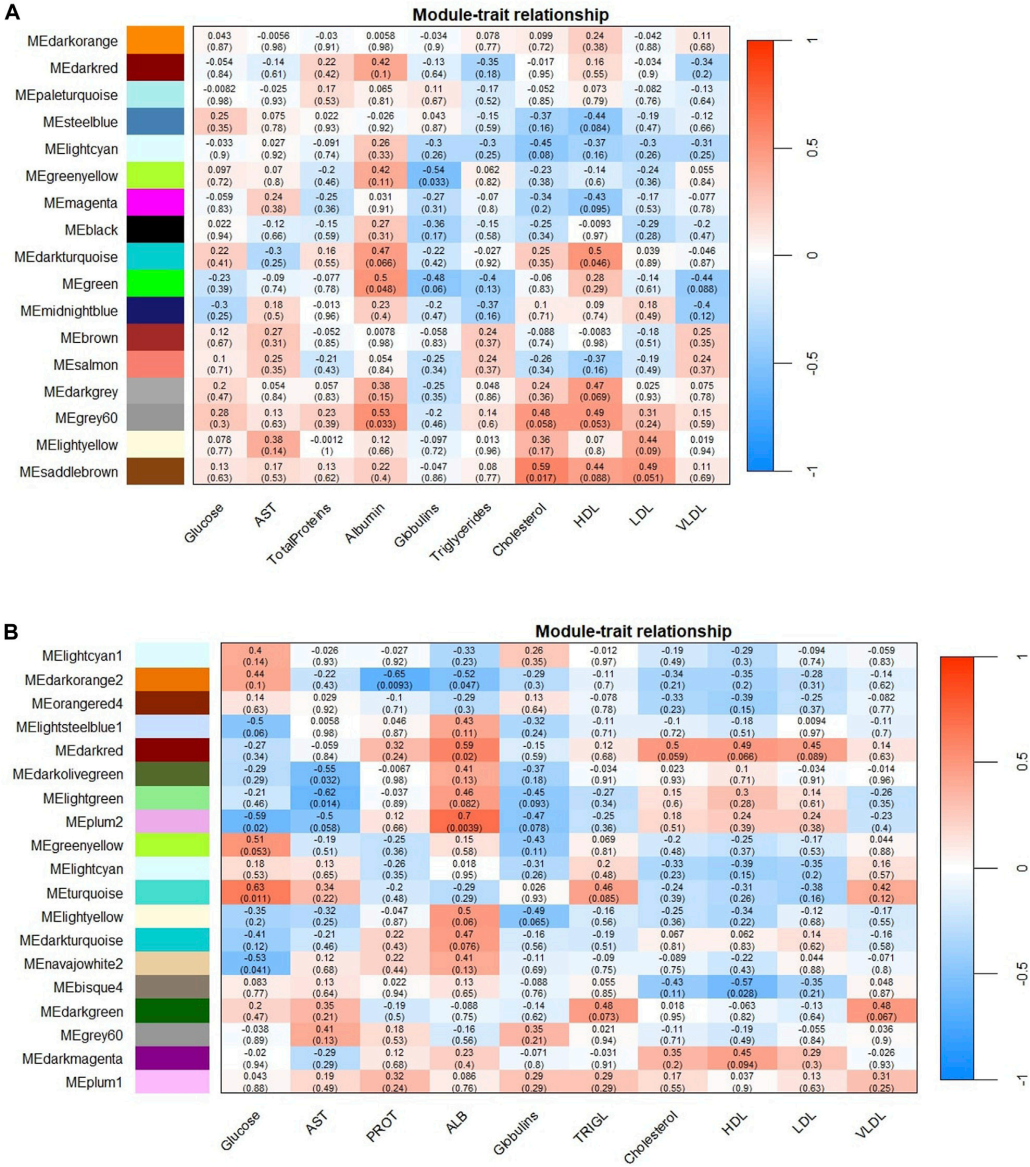
Module-trace associations between eigengene modules (ME) and the biochemical parameters studied. Panel **(A)** corresponds to SOY1.5 dietary treatment associations, while panel **(B)** corresponds to SOY3.0. Each row corresponds to an eigengene module, and each column to a biochemical parameter. Each cell contains the Pearson coefficient (number outside the parentheses) and the correction *p-*value (number in parentheses). The graphs are color-correlation coded according to the legend, where red represents a positive correlation and blue represents a negative correlation. AST: Aspartate aminotransferase; PROT: Total protein; ALB: Albumin, and TRIGL: Triglycerides.

The Pearson’s correlation coefficient between the ME and their respective variables illustrates the correlation between the module and the phenotypic information (Figure 1).

As shown in Figure 1, we identified 5 ME for SOY1.5 and 10 ME for SOY3.0 (*p-*value <0.05) in the gene co-expression network analysis performed. All nodes identified in each color are in Table 1. This suggests that the addition of 3% soybean oil to the diet may cause changes due to the difference in oils.

**TABLE 1:** Module Eigengene (ME) and nodes in groups SOY1.5 and SOY3.0.

ME SOY3.0 group	Nodes	ME SOY1.5 group	Nodes
*Lightcyan1*	101	*Darkorange*	170
*Darkorange2*	92	*Darkred*	211
*Orangered4*	114	*Paleturquoise*	85
*Lightsteelblue1*	101	*Steelblue*	121
*Darkred*	288	*Lightcyan*	273
*Darkolivegreen*	135	*Greenyellow*	464
*Lightgreen*	357	*Magenta*	511
*Plum2*	71	*Black*	618
*Greenyellow*	442	*Darkturquoise*	200
*Lightcyan*	408	*Green*	696
*Turquoise*	1227	*Midnightblue*	277
*Lightyellow*	343	*Brown*	1396
*Darkturquoise*	255	*Salmon*	393
*Navajowhite2*	56	*Darkgrey*	177
*Bisque4*	75	*Grey60*	251
*Darkgreen*	270	*Lightyellow*	246
*Grey60*	406	*Saddlebrown*	139
*Darkmagenta*	132		
*Plum1*	118		

In the SOY1.5 group, a significant association was observed between modules and traits such as albumin, cholesterol, globulin, and HDL (*p-*value <0.05). Specifically, albumin showed a positive correlation with both the *Green* module (*r* = 0.5) and the *Grey60* module (*r* = 0.53), while cholesterol was positively correlated with the *Saddlebrown* module (*r* = 0.59). Furthermore, HDL also showed a positive correlation with the *Darkturquoise* module (*r* = 0.5). Additionally, globulins showed a negative correlation with the *Greenyellow* module (*r* = −0.54). In SOY3.0, significant module-trait associations were found for glucose, AST, total proteins, albumin, and HDL across 10 modules (*p-*value <0.05). Glucose showed a negative correlation with the *Plum2* (*r* = −0.59) and *Navajowhite2* (*r* = −0.53) and a positive correlation with *Turquoise* (*r* = 0.63). AST was negative correlation with the *Darkolivegreen* (*r* = −0.55) and *Lightgreen* (*r* = −0.62). The *Darkorange2* was found to be associated with total proteins (*r* = −0.65). Albumin was positively correlated with the *Darkred* (r = 0.59) and *Plum2* (*r* = 0.7) modules, and negative correlated with the *Darkorange2* (*r* = −0.52). HDL was negatively correlated with the *Bisque4* module (*r* = −0.57).

As mentioned above, the identified correlations can be used to support in-depth studies on the effects of oil levels in pig diets and how they obtained gene interactions may play different roles in regulation.

### 3.2 Functional enrichment analysis of genes in relevant modules

We used Cytoscape to visualize networks with high weight nodes and edges. We have organized the results into separate topics for each phenotype, to provide a comprehensive understanding of the underlying gene interactions and regulatory mechanisms in response to soybean oil inclusion in the diet (SOY1.5 or SOY3.0).

### 3.3 Analysis of albumin-associated modules and their differential enrichment across diets

For albumin phenotype, two modules were identified for SOY1.5 and three for SOY3.0. Among these, the *Grey60* module in SOY1.5 was selected, containing nodes and edges with a weight >0.19. The *Grey60* module was found to be associated with the albumin phenotype and comprised 755 nodes and 280,021 edges. After applying filtering, the resulting network was 112 nodes and 357 edges (Supplementary Table S3A) incorporating genes such as 3-hydroxyacyl-CoA dehydratase 1 (*HACD1*), glycogen phosphorylase, muscle-associated (*PYGM*), histone deacetylase 6 (*HDAC6*), formin-binding protein 4 (*FNBP4*), and protein phosphatase 1 regulatory subunit 12B (*PPP1R12B*), among others.

The enriched GO terms included histone acetyltransferase complex (GO:0000123) with genes such as acrosin binding protein (*ACRBP*) and *OGT* gene. Another enriched term was regulation of transcription (GO:0006357; GO:0006355) with genes like bromodomain-containing 2 (*BRD2*), zinc finger protein 630 (*ZNF630*), cyclin D binding myb-like transcription factor 1 (*DMTF1*), and notch receptor 3 (*NOTCH3*). Additionally, the DNA binding term (GO:0003677) was enriched and included genes such as methyl-CpG binding domain protein 6 (*MBD6*), nucleic acid binding protein 1 (*NABP1*), among others.

**FIGURE 2:**
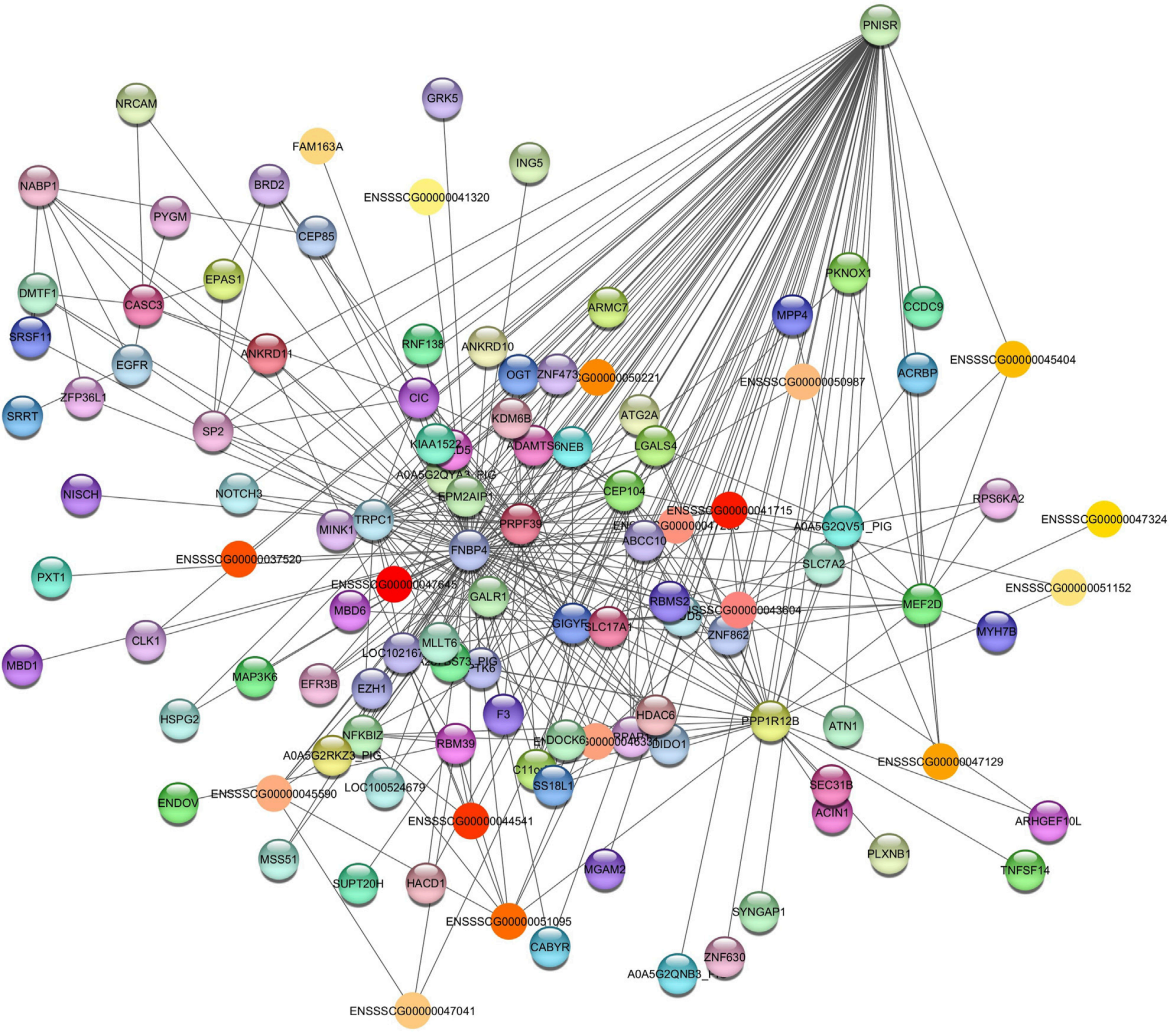
Network view highlighting interactions of the hub gene *PNISR*. Interaction networks within the liver tissue from pigs fed a diet with 1.5% soybean oil inclusion (SOY1.5) were constructed using Cytoscape, followed by the stringApp plugin. The network associated with the *Grey60* module is co-expressed with the albumin phenotype. Colored nodes represent genes/proteins included in the query.

The hub gene identified was PNN Interacting Serine and Arginine Rich Protein (*PNISR*) for the *Grey60* module (Figure 2). *PNISR* is a serine-arginine-rich protein that plays a role in the pre-mRNA splicing machinery (Zappaterra et al., 2020).

Regarding the SOY1.5 group, the *Green* module, similar to the *Grey60* module, showed a moderate correlation with the albumin phenotype. This module consists of 867 nodes and 373,625 edges. To ensure the relevance of our findings, we applied a filtering criterion with a weight threshold >0.18, resulting in a refined network comprising 95 nodes and 252 edges (Supplementary Table S3B). Genes included in this network are interleukin 16 (*IL16*), interleukin 10 receptor subunit alpha (*IL10RA*), C-X-C motif chemokine ligand 13 (*CXCL13*), FXYD domain containing ion transport regulator 2 (*FXYD2*).

The network showed enrichments related to GO terms, including immune response (GO:0006955) with genes such as C-C motif chemokine ligand 5 (*CCL5*), lymphocyte antigen 86 (*LY86*), and transport vesicle membrane (*CD74*), among others. Another significant GO term was positive regulation of ERK1 and ERK2 cascade (GO:0070374) with genes like CD4 molecule (*CD4*)*,* caveolae associated (*CAVIN3*), arrestin beta 1 (*ARRB1*), arrestin beta 2 (*ARRB2*), and *CD74*. Additionally, negative regulation of NF-kappaB transcription factor activity (GO:0032088) was associated with genes such as *ARRB1*, *ARRB2*, and coronin 1A (*CORO1A*), along with other relevant terms.

Moreover, we observed significant enrichments in KEGG pathways, including hematopoietic cell lineage (ssc04640) with genes such as CD3 epsilon subunit of T-cell receptor complex (*CD3E*), *CD4*, MHC class II DR-alpha (*HLA-DRA*), and SLA-DQ beta1 domain (*SLA-DQB1*). The Th1 and Th2 cell differentiation pathway (ssc04658) exhibited the same genes. Additionally, the type I diabetes mellitus pathway (ssc04940) showed associations with genes such as *HLA-DRA*, *SLA-DQA*, *SLA-DQB1*, and MHC class II, DM beta (*SLA-DMB*), along with other pathways. In this context, the module displays genes with important relationships to immune response, cell signaling and disease.

**FIGURE 3:**
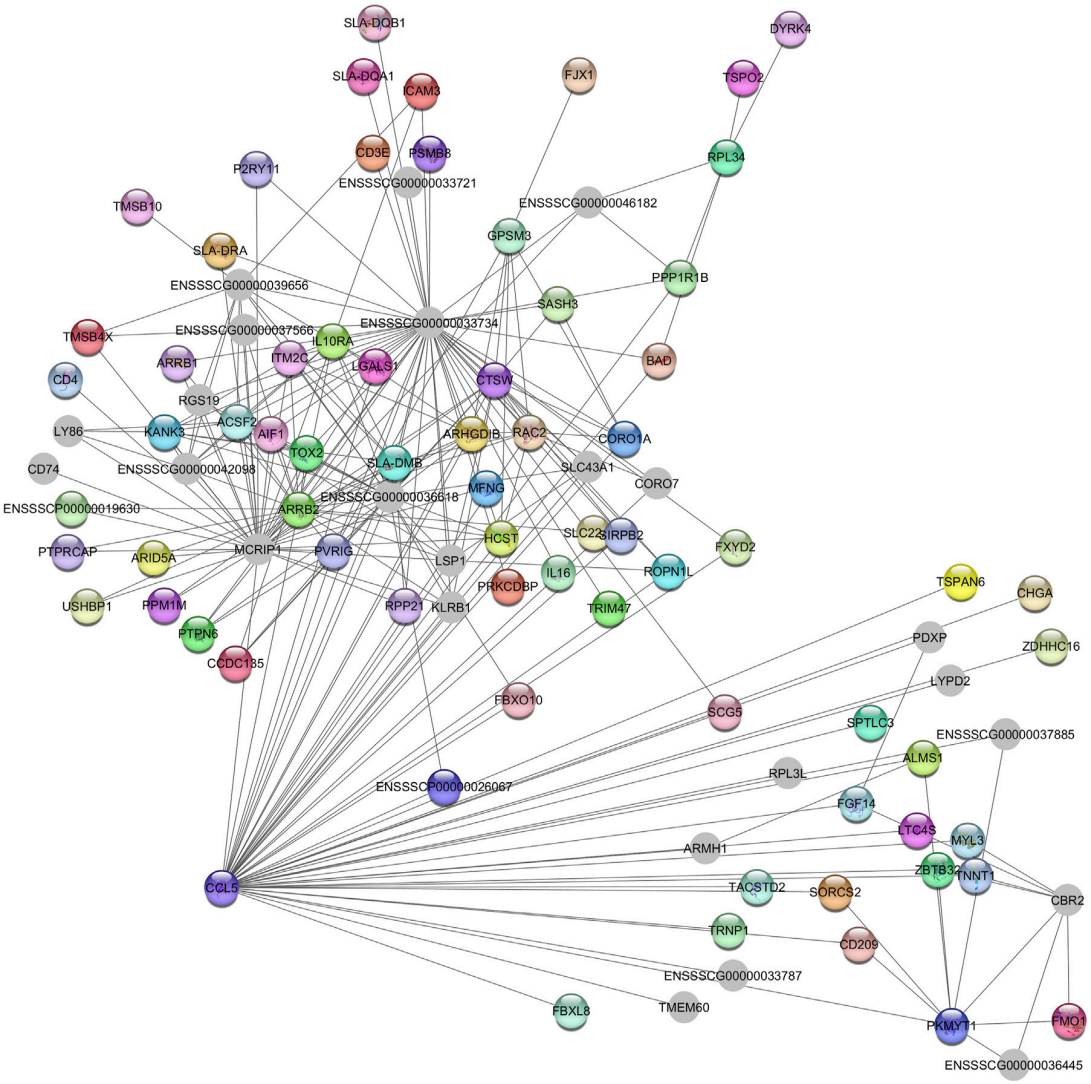
Network view highlighting interactions of the hub gene *CCL5*. Interaction networks within the liver tissue from pigs fed a diet with 1.5% soybean oil inclusion (SOY1.5) were constructed using Cytoscape, followed by the stringApp plugin. The network associated with the *Green* module is co-expressed with the albumin phenotype. Colored nodes represent genes/proteins included in the query.

The hub gene identified in the *Green* module is *CCL5*, which is associated with immunoregulatory and inflammatory processes (Figure 3) (O’Leary et al., 2016).

For the SOY3.0 group, the *Darkorange2, Darkred, and Plum2* modules were identified as associated with albumin.

**FIGURE 4:**
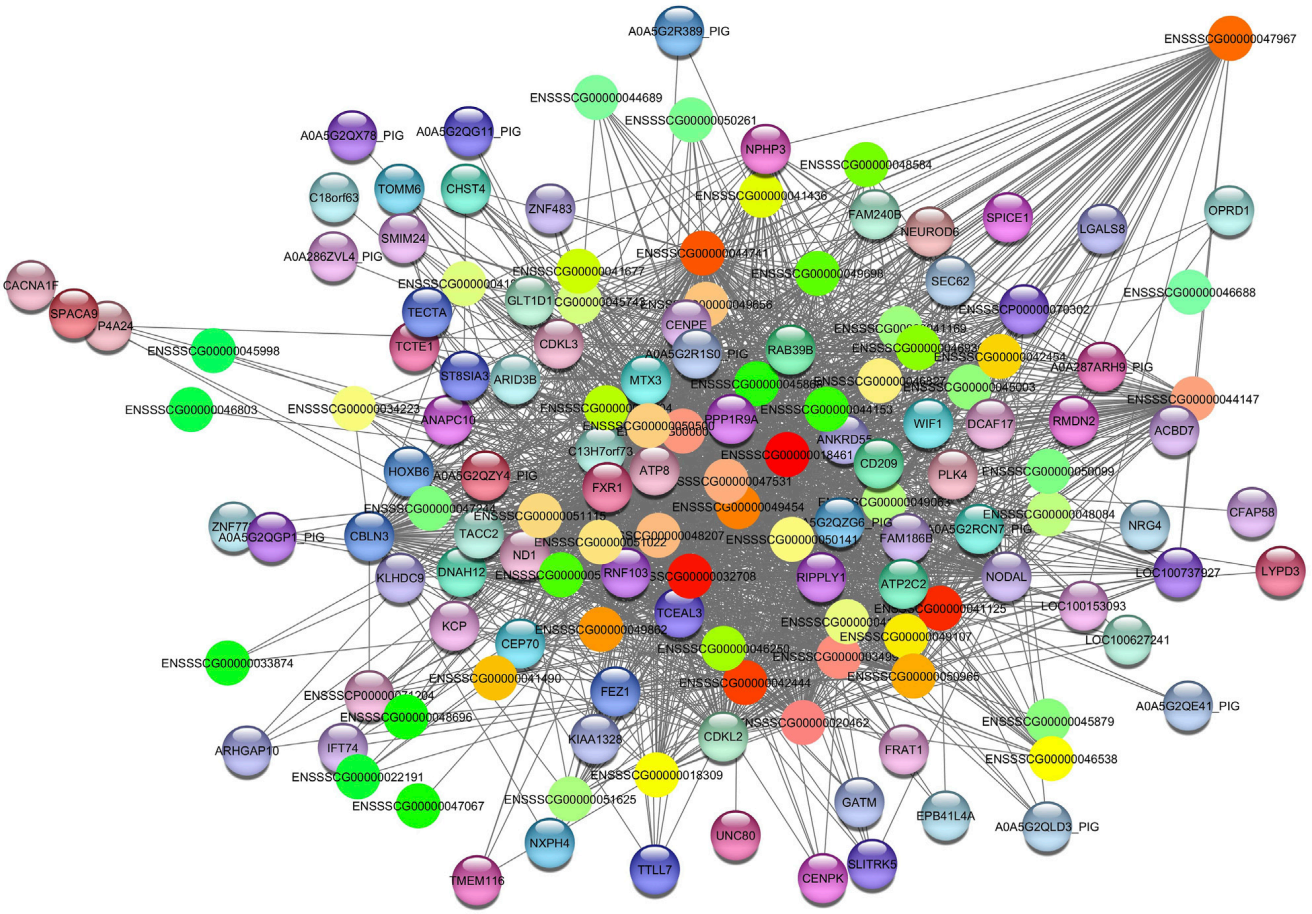
Network view highlighting interactions of the hub gene *ENSSCG00000047967*. Interaction networks within the liver tissue from pigs fed a diet with 3% soybean oil inclusion (SOY3.0) were constructed using Cytoscape, followed by the stringApp plugin. The network associated with the *Darkred* module is co-expressed with the albumin phenotype. Colored nodes represent genes/proteins included in the query.

The *Darkred* module consists of 288 nodes and 35,408 edges. To ensure the relevance of our findings, we applied a filtering criterion with a weight threshold >0.14, resulting in a refined network comprising 142 nodes and 2047 edges (Supplementary Table S4A). The enrichment pathways of the *Darkred* module, such as the (ssc05022) pathways of neurodegeneration—multiple diseases, with genes such as calcium voltage-gated channel subunit alpha1 F (*CACNA1F)*, NADH dehydrogenase subunit 1 (*ND1)*, ATP synthase F0 subunit 8 (*ATP8*), dynein axonemal heavy chain 12 (*DNAH12)*, RAB39B, member of the RAS oncogene family (*RAB39B*) and *FRAT1* (FRAT regulator of WNT signaling pathway 1). Additionally, Alzheimer’s disease pathway (ssc05010) was also identified (Table 2). The hub gene identified in this module is *ENSSCG00000047967* (Figure 4).

**TABLE 2:** Pathways enriched in the *Darkred* module for SOY3.0.

Top 10 pathways enriched in the *Darkred* module for SOY3.0	*p*-value	Genes
ssc05022:Pathways of neurodegeneration - multiple diseases	0.0028	ENSSSCG00000018065, ENSSSCG00000024233, ENSSSCG00000012298, ENSSSCG00000020990, ENSSSCG00000032821, ENSSSCG00000018080
GO:0007368∼determination of left/right symmetry	0.0068	ENSSSCG00000030998, ENSSSCG00000011632, ENSSSCG00000005124
GO:0005813∼centrosome	0.0126	ENSSSCG00000010614, ENSSSCG00000024663, ENSSSCG00000026746, ENSSSCG00000008970, ENSSSCG00000015212, ENSSSCG00000005124
GO:0005856∼cytoskeleton	0.0159	ENSSSCG00000029860, ENSSSCG00000023728, ENSSSCG00000015329, ENSSSCG00000010614, ENSSSCG00000037530
GO:0007018∼microtubule-based movement	0.0274	ENSSSCG00000001705, ENSSSCG00000023296, ENSSSCG00000020990
GO:0038092∼nodal signaling pathway	0.0321	ENSSSCG00000039399, ENSSSCG00000030998
GO:0051382∼kinetochore assembly	0.0349	ENSSSCG00000016946, ENSSSCG00000023296
ssc05014:Amyotrophic lateral sclerosis	0.0423	ENSSSCG00000018065, ENSSSCG00000024233, ENSSSCG00000020990, ENSSSCG00000018080
ssc05010:Alzheimer disease	0.0483	ENSSSCG00000018065, ENSSSCG00000012298, ENSSSCG00000032821, ENSSSCG00000018080

**FIGURE 5:**
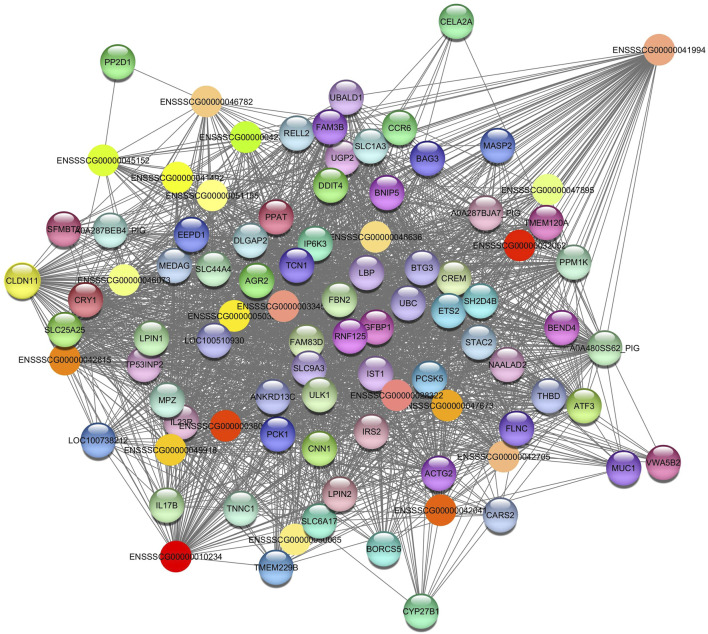
Network view highlighting interactions of the hub gene ENSSSCG00000041994. Interaction networks within the liver tissue from pigs fed a diet with 3% soybean oil inclusion (SOY3.0) were constructed using Cytoscape, followed by the stringApp plugin. The network associated with the *Darkorange2* module is co-expressed with the total protein and albumin phenotypes. Colored nodes represent genes/proteins included in the query.

*Darkorange2* module was identified in total protein and albumin. It showed a negative correlation in both phenotypes. For the *Darkorange2*, the hub gene identified was *ENSSSCG00000041994* (Figure 5). The enriched pathways are related to fatty acid catabolic process (GO:0009062); gluconeogenesis (GO:0006094); response to insulin (GO:0032868); among others (Table 3). This module consists of 92 nodes and 3015 edges. To ensure the relevance of our findings, we applied a filtering criterion with a weight threshold >0.03, resulting in a refined network comprising 91 nodes and 2312 edges (Supplementary Table S4B).

**TABLE 3:** Pathways enriched in the *Darkorange2* module for SOY3.0.

Pathways enriched in the *Darkorange2* module for SOY3.0	*p*-value	Genes
GO:0006094∼gluconeogenesis	0.0107	ENSSSCG00000015595, ENSSSCG00000007507, ENSSSCG00000000164
GO:0008104∼protein localization	0.0292	ENSSSCG00000002743, ENSSSCG00000022099, ENSSSCG00000009742
GO:0008195∼phosphatidate phosphatase activity	0.0434	ENSSSCG00000008624, ENSSSCG00000038494
GO:0009062∼fatty acid catabolic process	0.0242	ENSSSCG00000008624, ENSSSCG00000038494
GO:0014823∼response to activity	0.0272	ENSSSCG00000005636, ENSSSCG00000000164
GO:0032868∼response to insulin	0.0057	ENSSSCG00000016728, ENSSSCG00000003459, ENSSSCG00000000164
GO:0032869∼cellular response to insulin stimulus	0.0119	ENSSSCG00000008624, ENSSSCG00000038494, ENSSSCG00000007507
GO:0042594∼response to starvation	0.0479	ENSSSCG00000007507, ENSSSCG00000009742
GO:0071549∼cellular response to dexamethasone stimulus	0.0391	ENSSSCG00000007507, ENSSSCG00000031888
ssc04150:mTOR signaling pathway	0.0199	ENSSSCG00000008624, ENSSSCG00000038494, ENSSSCG00000031888, ENSSSCG00000009742

**FIGURE 6:**
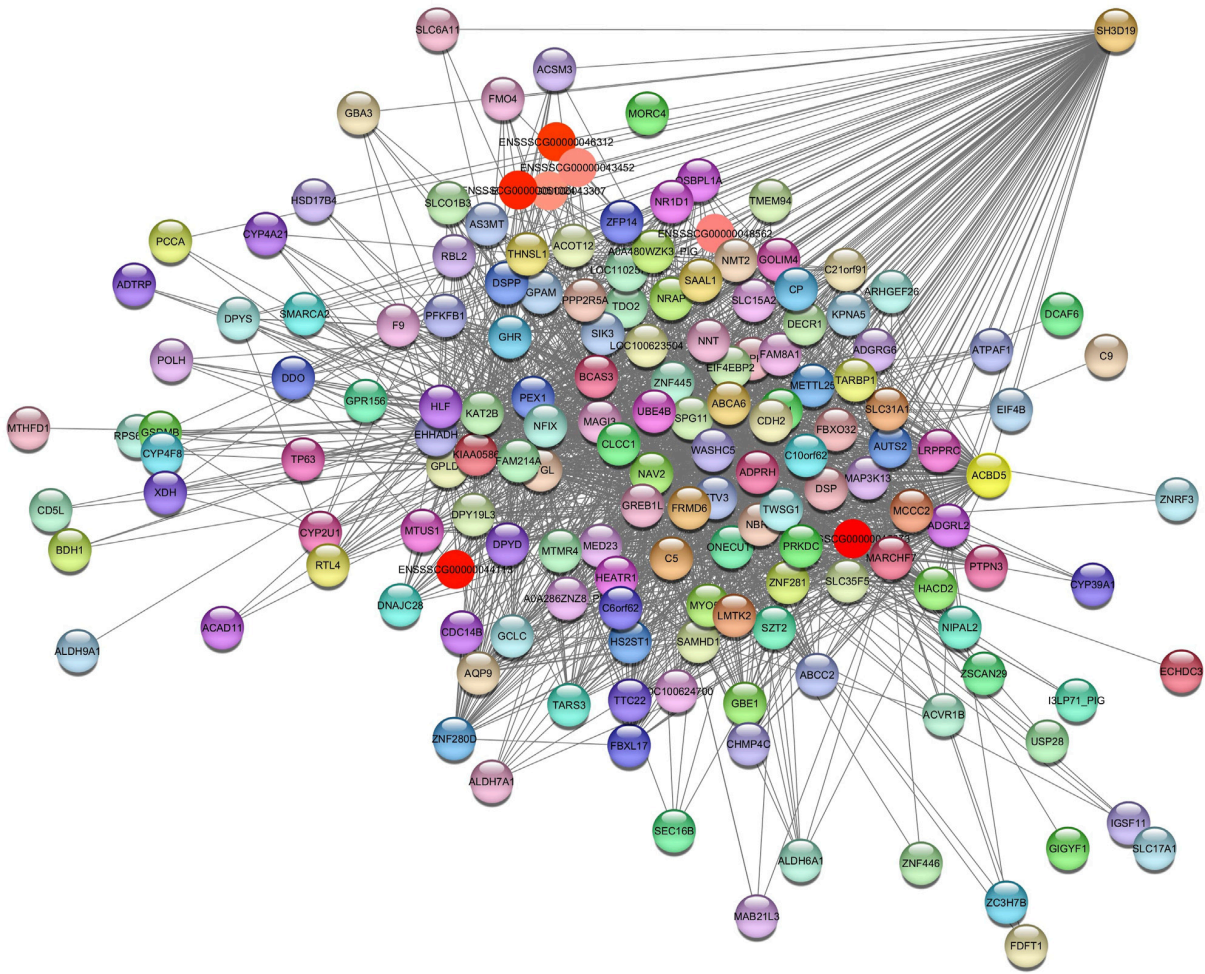
Network view highlighting interactions of the hub gene *SH3D19*. Interaction networks within the liver tissue from pigs fed a diet with 3% soybean oil inclusion (SOY3.0) were constructed using Cytoscape, followed by the stringApp plugin. The network associated with the *Plum2* module is co-expressed with the glucose and albumin phenotype. Colored nodes represent genes/proteins included in the query.

For the *Plum2* module (Supplementary Table S4C), pathways were enriched, such as insulin-like growth factor receptor signaling pathway (GO:0048009), valine, leucine and isoleucine degradation (ssc00280), metabolic pathways (ssc01100), fatty acids degradation (ssc00071), acyl-CoA metabolic process (GO:0006637), and ssc04976 (bile secretion) (Supplementary Table S5). The hub gene identified is SH3 Domain Containing 19 (*SH3D19)* (Figure 6).

This module is related to glucose and albumin. It has a strong positive correlation with albumin and a negative correlation with glucose.

### 3.4 Hub genes and pathways related to AST

**FIGURE 7:**
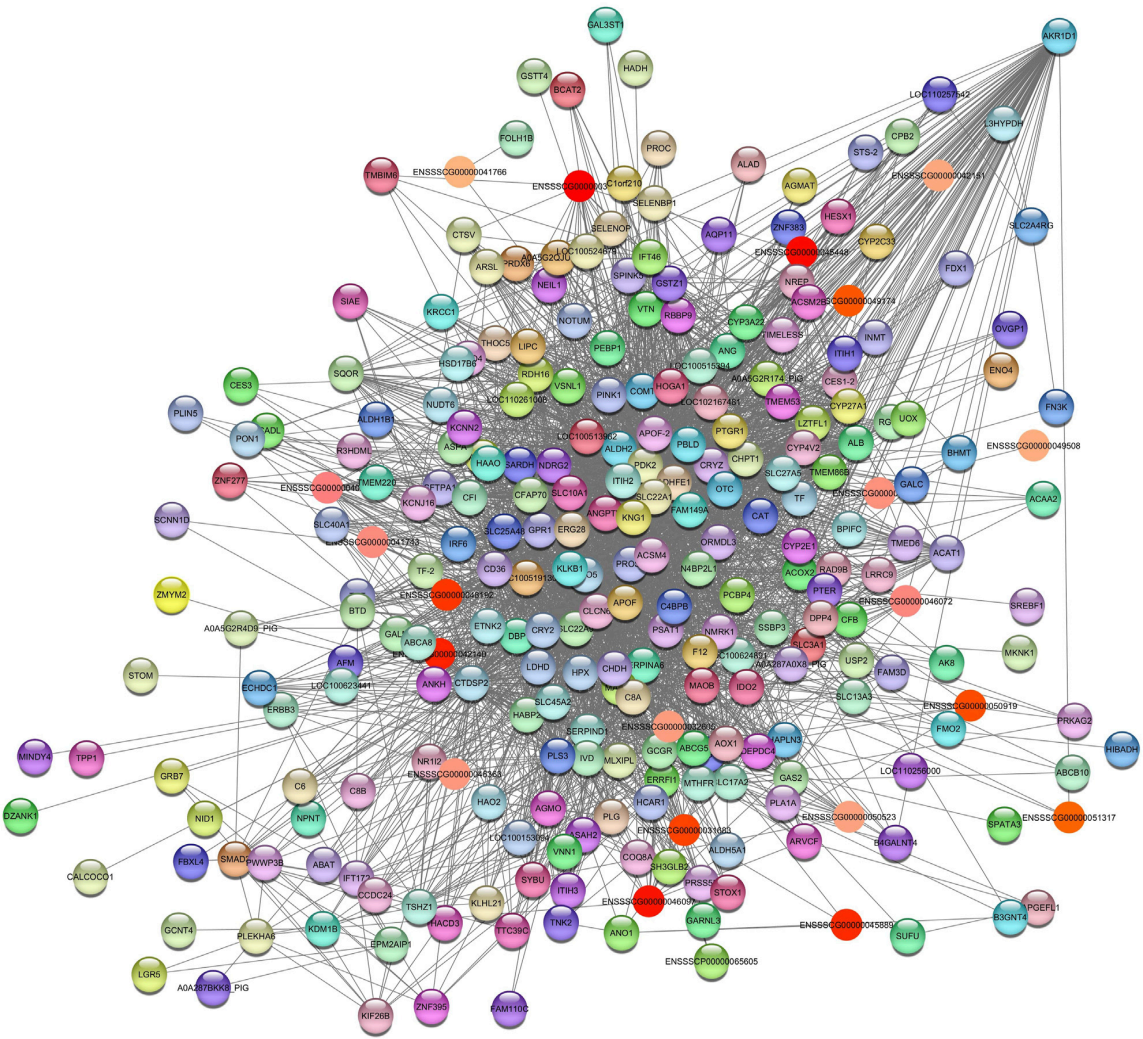
Network view highlighting interactions of the hub gene *AKR1D1*. Interaction networks within the liver tissue from pigs fed a diet with 3% soybean oil inclusion (SOY3.0) were constructed using Cytoscape, followed by the stringApp plugin. The network associated with the *Lightgreen* module is co-expressed with the AST phenotype. Colored nodes represent genes/proteins included in the query (small nodes indicate proteins with unidentified 3D structure, large nodes indicate those with known structures).

For AST, we identified the *Darkolivegreen* and *Lightgreen* modules in SOY3.0. After filtering for values >0.05, the network for *Darkolivegreen* module consisted of 6595 nodes and 2698 edges (Supplementary Table S6A). In the *Lightgreen* module, after applying a filter >0.10, the network retained 237 nodes and 2158 edges (Supplementary Table S6B). In the *Lightgreen* module, pathways were identified as (GO:0006629) lipid metabolic process with the genes *LIPC, FMO5, COMT, PLA1A, AOX1, ACSM4, SLC27A5* and *BCAT2*. Additionally, pathways such a cholesterol homeostasis (GO:0042632); PPAR signaling pathway (ssc03320); fatty acid metabolism (ssc01212); gene expression (GO:0010467); fatty acid binding (GO:0005504); metabolic pathways (ssc01100) were enrichment. The hub gene aldo-keto reductase family 1 member D1 (*AKR1D1)* was identified (Figure 7).

Deficiency of the enzyme encoded by this gene may contribute to liver dysfunction (Stelzer et al., 2016).

**FIGURE 8:**
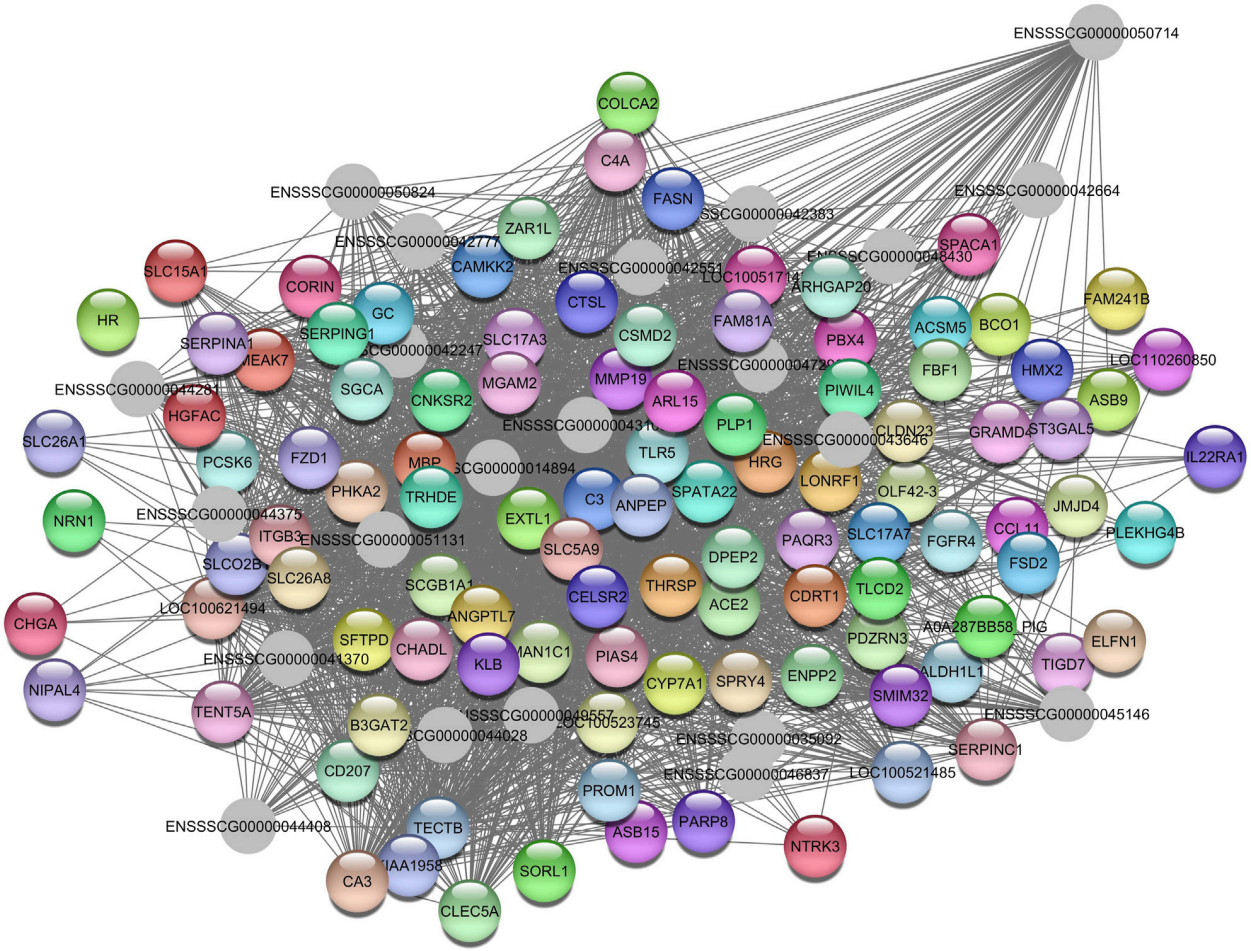
Network view highlighting interactions of the hub gene *ENSSSCG00000050714*. Interaction networks within the liver tissue from pigs fed a diet with 3% soybean oil inclusion (SOY3.0) were constructed using Cytoscape, followed by the stringApp plugin. The network associated with the *Darkolivegreen* module is co-expressed with the AST phenotype. Colored nodes represent genes/proteins included in the query.

Regarding the *Darkolivegreen* module, the hub gene *ENSSSCG00000050714* was identified (Figure 8), and the enriched pathways were inflammatory response (GO:0006954); metabolic pathways (ssc01100); regulation of triglyceride biosynthetic process (GO:0010866); innate immune response (GO:0045087); and transmembrane transport (GO:0055085).

### 3.5 Identifying modules and key genes associated with globulins

The *Greenyellow* module was found to be moderately associated with the globulin’s phenotype in SOY1.5. The network constructed from this module consisted of 466 nodes and 106,695 edges. After applying a filter to remove edges with a weight less than 0.17, the resulting network contained 144 nodes and 554 edges (Supplementary Table S7), which included genes such as OTU domain-containing ubiquitin aldehyde-binding protein 1 (*OTUB1)*, leukotriene A4 hydrolase (*LTA4H*); niban apoptosis regulator 3 (*NIBAN3*); and eukaryotic translation initiation factor 4H (*EIF4H*).

**FIGURE 9:**
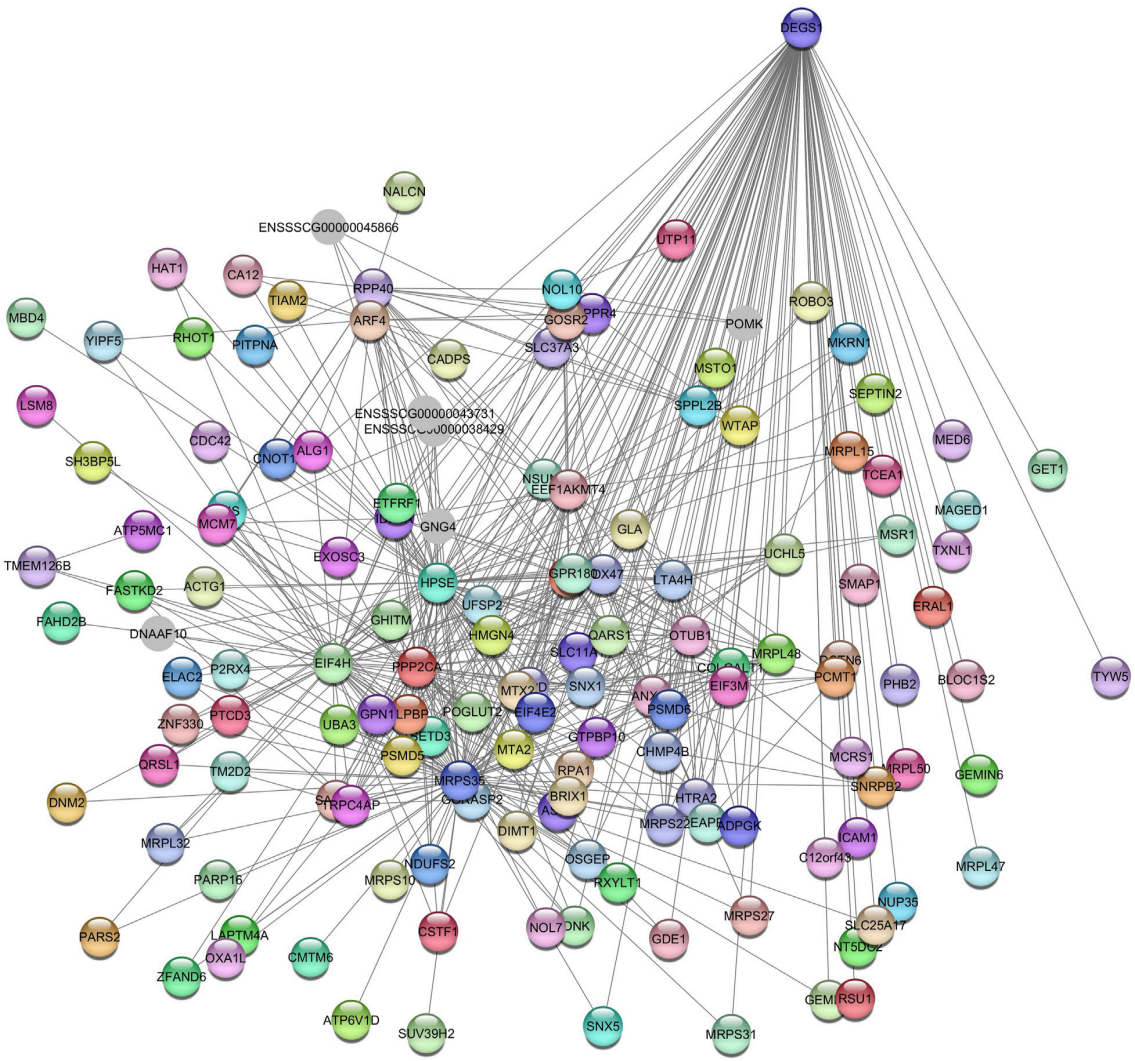
Network view highlighting interactions of the hub gene *DEGS1*. Interaction networks within the liver tissue from pigs fed a diet with 1.5% soybean oil inclusion (SOY1.5) were constructed using Cytoscape, followed by the stringApp plugin. The network associated with the *Greenyellow* module is co-expressed with the globulin phenotype. Colored nodes represent genes/proteins included in the query.

Enriched KEGG pathways included endocytosis (ssc04144), amyotrophic lateral sclerosis (ssc05014), and aminoacyl-tRNA biosynthesis (ssc00970), among others. The GO terms enriched included GTP binding (GO:0005525), rRNA methylation (GO:0031167), translation initiation factor activity (GO:0003743), and golgi apparatus (GO:0005794), among others. The gene identified as the hub gene is Delta 4-Desaturase, Sphingolipid 1 *(DEGS1)* (Figure 9).

### 3.6 Identification of modules associated with cholesterol, and HDL

In the SOY1.5 group, the *Saddlebrown* module was associated with total cholesterol. The network contained 139 nodes and 9,580 edges. After filtering with a weight threshold of >0.13, the network was reduced to 141 nodes and 752 edges. Genes represents in this module include TNF receptor superfamily member 11b (*TNFRSF11B*), Fos proto-oncogene, AP-1 transcription factor subunit (*FOS*), solute carrier family 2 member 3 (*SLC2A3*), mitogen-activated protein kinase kinase kinase 8 (*MAP3K8*), vascular cell adhesion molecule 1 (*VCAM1*), and vascular endothelial growth factor C (*VEGFC*), among others.

The cholesterol-related hub gene identified in the *Saddlebrown* module is synaptonemal complex protein 3 *(SYCP3).* Suppression of SYCP3 affects the expression of genes associated with lipid metabolism. However, in the SOY1.5 group, the MM vs*.* GS for the module did not show a *p*-value <0.05.

On the other hand, HDL had modules in both SOY1.5 and SOY3.0. The *Darkturquoise* module was identified in SOY1.5, while the *Bisque4* module were found in SOY3.0. The *Darkturquoise* module in the network consisted of 63 nodes and 252 edges (Supplementary Table S8A). Notable genes within this module include apolipoprotein E (*APOE*), apolipoprotein A2 (*APOA2*), fatty acid binding protein 1 (*FABP1*), and transcription factor like 5 (*TCFL5*).

The network was enriched for cholesterol metabolism (ssc04979), metabolic pathways (ssc01100). The GO terms such as high-density lipoprotein particle clearance (GO:0034384), tetrahydrobiopterin biosynthetic process (GO:0006729), positive regulation of cholesterol esterification (GO:0010873), high-density lipoprotein particle assembly (GO:0034380), phospholipid efflux (GO:0033700), low-density lipoprotein particle remodeling (GO:0034374), reverse cholesterol transport (GO:0043691), high-density lipoprotein particle remodeling (GO:0034375), mitochondrial membrane (GO:0031966), chylomicron (GO:0042627), and very-low-density lipoprotein particle (GO:0034361), among others.

**FIGURE 10:**
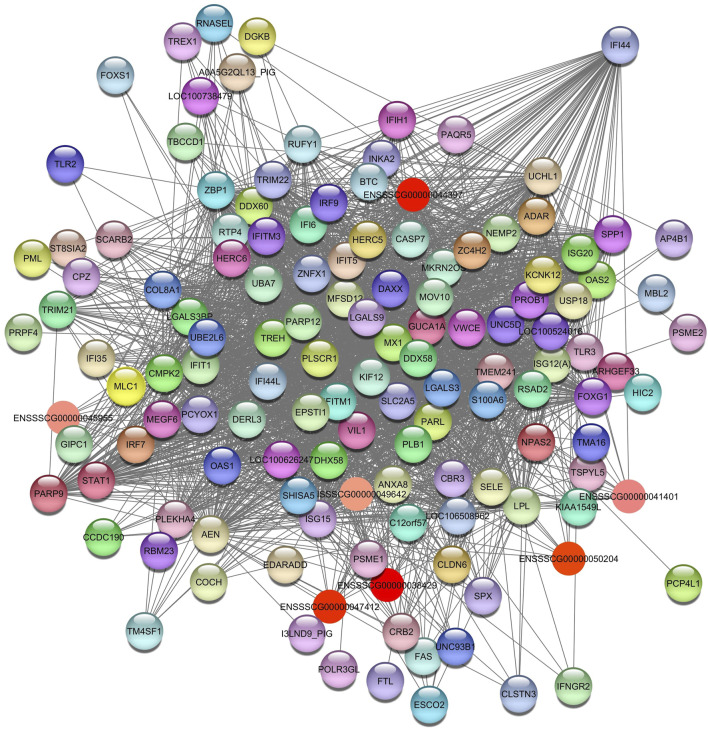
Network view highlighting interactions of the hub gene *IF144*. Interaction networks within the liver tissue of pigs fed a diet with 3.0% soybean oil inclusion (SOY3.0) were constructed using Cytoscape, followed by the stringApp plugin. The network associated with the *Bisque4* module is co-expressed with the HDL phenotype. Colored nodes represent genes/proteins included in the query.

Relevant genes and pathways associated with the HDL in the liver were identified for SOY3.0 (Supplementary Table S8B). The enrichment analysis identified pathways such as positive regulation of I-kappaB kinase/NF-kappaB signaling (GO:0043123); response to interferon-gamma (GO:0034341); toll-like receptor signaling pathway (GO:0002224); ubiquitin protein ligase binding (GO:0031625); interleukin-27-mediated signaling pathway (GO:0070106); immune system process (GO:000237); and positive regulation of interleukin-6 production (GO:0032755). The hub gene identified in this pathway was interferon induced protein 44 (*IF144)* (Figure 10).

### 3.7 Identification of modules and key genes associated with glucose

Three modules were identified for glucose*: Plum2, Turquoise, Navajowhite2* for SOY3.0. As previously observed, the *Plum2* module with the hub gene *SH3D19*, just as in the glucose phenotype.

**FIGURE 11:**
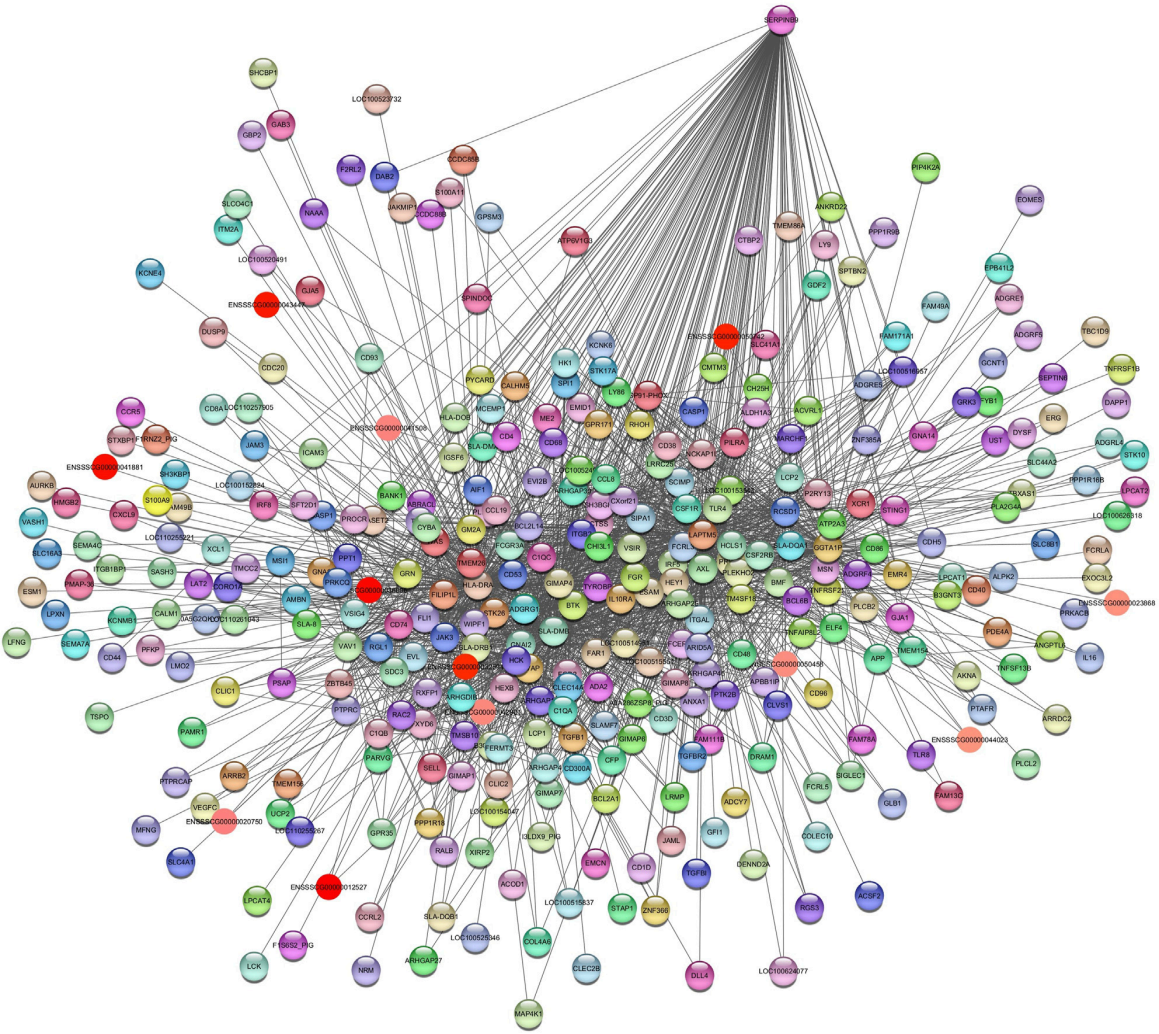
Network view highlighting interactions of the hub gene *SERPINB9*. Interaction networks within the liver tissue of pigs fed a diet with 3.0% soybean oil inclusion (SOY3.0) were constructed using Cytoscape, followed by the stringApp plugin. The network associated with the *Turquoise* module is co-expressed with the glucose phenotype. Colored nodes represent genes/proteins included in the query.

In the *Turquoise* module (SSupplementary Table S9A), the enrichment pathways included immune response (GO:0006955), inflammatory response (GO:0006954), response to cholesterol (GO:0070723), negative regulation of I-kappaB kinase/NF-kappaB signaling (GO:0043124), positive regulation of interleukin-4 production (GO:0032753), positive regulation of interleukin-2 production (GO:0032743), positive regulation of angiogenesis (GO:0045766), and Th1 and Th2 cell differentiation (ssc04658). The hub gene identified is serpin family B member 9 (*SERPINB9)* (Figure 11).

For the *Navajowhite2* module (Supplementary Table S9B), we identified enriched pathways as spliceosome (ssc03040); NF-kappaB binding (GO:0051059); RNA binding (GO:0003723); RNA helicase activity (GO:0003724).

**FIGURE 12:**
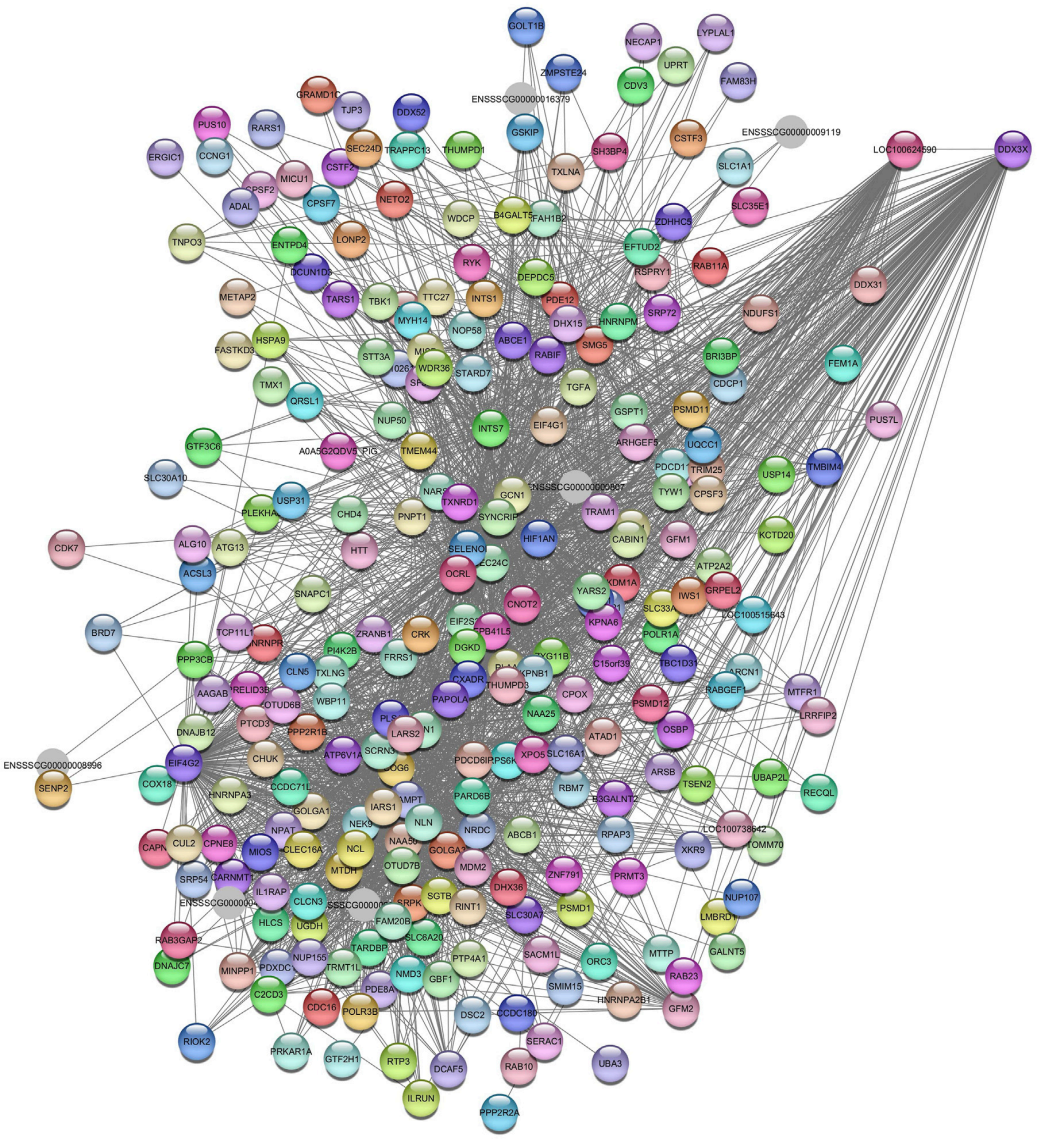
Network view highlighting interactions of the hub gene *DDX3X*. Interaction networks within the liver tissue of pigs fed a diet with 3.0% soybean oil inclusion (SOY3.0) were constructed using Cytoscape, followed by the stringApp plugin. The network associated with the *Najowhite2* module is co-expressed with the glucose phenotype. Colored nodes represent genes/proteins included in the query.

The DEAD-box helicase 3 X-linked (*DDX3X*) gene was identified as a hub gene in the *Navajowhite2* module (Figure 12).

## 4 Discussion

The WGCNA algorithms, according to Xing et al. (2021), attempt to build a network with correlation values that match the properties of a scale-free network because they have biological significance. Thus, in this study, we identified the gene modules and the main signaling pathways that influence the blood serum parameters of pigs fed different levels of soybean oil from the networks constructed by the WGCNA. The variation in the number of modules identified in both SOY1.5 and SOY3.0 can be attributed to differences in the genes expressed in the liver, possibly due to regulatory mechanisms in response to oil intake. This would help to explain the differences in module content and enrichment results between the groups. In addition, these results support that regulation of gene expression plays a role in influencing pathways that may affect traits related to blood serum parameters.

A higher number of ME was observed in the SOY3.0 group. In our previous study, we observed differences in albumin levels between the SOY1.5 and SOY3.0 diet groups, with higher values observed in the SOY1.5 group (Fanalli et al., 2022b). Our results illustrate the changes in co-expression in the SOY1.5 and SOY3.0 groups in the diet, furthering our understanding of metabolic processes and their relevance to disease. These results are consistent with our previous findings indicating changes in gene expression when 3% soybean oil was added to the diet, showing differential expression (Fanalli et al., 2022c).

### 4.1 Co-expressed modules related to albumin

By examining the co-expression of albumin-related genes, we can understand the potential mechanisms and processes that are regulated by FA in the liver. Albumin is produced by the liver and is associated with a wide range of important physiological functions, such as immunomodulation and antioxidant effects (Spinella et al., 2016). Albumin may serve as an important modulator of innate immune responses to systemic inflammation (Spinella et al., 2016). Immune-related diseases are linked to the body’s defense against infection. In this context, the inclusion of immune nutrients, such as FA, has the potential to alter the body’s inflammatory and immune responses, as FA is involved in modulating macrophage functions (Radzikowska et al., 2019). This has been attributed to the immunomodulatory effects of PUFA, particularly those from the omega-3 series, which can influence clinical outcomes by modulating the immune system (Miles and Calder, 2015; Fanalli et al., 2022b). In inflammatory conditions, omega-3 FA can restore impaired barrier function and reduce the pro-inflammatory mediator production (Radzikowska et al., 2019).

Albumin is used as an indicator to evaluate dysfunction in the liver, pancreas, and overall nutritional status. It is more susceptible to reactive oxygen species (ROS) compared to other proteins, and the proportion of oxidized albumin serves as a marker for the degree of oxidative stress associated with various pathological conditions (Belinskaia et al., 2021). FA, especially PUFA, plays an important role in regulating the antioxidant properties of albumin, with PUFA significantly contributing to the pool of oxidizable biological compounds in plasma. In addition, these biomarkers associated with oxidative stress may hold prognostic and therapeutic significance for acute-on-chronic liver failure *(ACLF)*. The gene *CCL5*, identified in this context, is correlated with immune response metabolic processes and immune response (Zhu and Jiang, 2023).

The hub gene identified in our study in the SOY1.5 group, that correlates with albumin, is *CCL5* (RANTES) in diabetes-related renal pathophysiology, and serum albumin may play a relevant role (Nakajima et al., 2003). Furthermore, omega-6 and omega-3 have been observed in FA-related studies to play a role in regulating inflammatory responses through nuclear receptors that influence gene expression (Ogłuszka et al., 2017). In a study investigating the effects of diet on gene expression in pig muscle, an increase in dietary PUFA was related with a decrease in the expression of several chemokines, including CCL5. These chemokines are known to attract macrophages and monocytes to sites of inflammation, and the authors suggested that this decrease in the expression of these chemokines could be related to a reduction (Ogłuszka et al., 2017). This confirms our previous results where we observed greater PUFA deposition in the liver in the SOY1.5 group. Dietary changes can modulate gene expression, affecting inflammation, lipid metabolism and homeostasis.

On the other hand, in the SOY3.0 group, we observe more albumin-related and neurodegenerative disease-related cluster groups such as *Darkred.* Polyunsaturated fatty acids, including those of the omega-3 and omega-6 series, have been shown to improve cognitive function in individuals with neurodegenerative diseases (Avallone et al., 2019). These FA achieve this by modulating cellular properties and physiological processes (Avallone et al., 2019). Consequently, diets supplemented with PUFA-rich vegetable oils, such as rapeseed and soybean oil, may offer benefits to consumers. Fanalli et al. (2022c), who investigated differentially expressed genes in the same pig population, identified genes associated with network maps related to neurodegenerative diseases.

Some of the GO terms and KEGG pathways we identified in this study are enriched to general metabolism, human diseases such as neurodegenerative and infectious diseases, and the immune system. This demonstrates the importance of the study and its relevance to biological processes. In addition, related KEGG pathways Alzheimer’s disease and neurodegeneration are specifically associated with albumin.

### 4.2 Co-expressed modules related to glucose

*S*ignificant changes observed in the SOY3.0 group could potentially have a more profound effect on gene expression and regulatory networks, leading to a broader effect on lipid and glucose metabolism in porcine liver tissue. The difference in co-expression observed in relation to glucose may be related to the regulation of angiogenesis, suggesting a specific response to the diet with a higher concentration of soybean oil (SOY3.0). According to Fernandez and West, (2005), omega-3 are involved in reducing plasma TG by decreasing lipogenesis and VLDL secretion.

Significantly, the SOY3.0 group showed increased accumulation of OA. Both MUFA and PUFA have been implicated in the regulation of key regulators of hepatic gene transcription. These FA affect transcription factors that play a role in influencing the expression of genes central to glycolysis, *de novo* fatty acid synthesis, and FA oxidation. This regulatory effect on lipid metabolism can be either direct or indirect (Gnoni et al., 2010).

### 4.3 Co-expressed modules related to HDL

We have identified *APOE* associated with the SOY1.5 group co-expressed with HDL. In a study in which the *APOE* gene was knockout in Bama miniature pigs using the CRISPR-associated protein 9 (CRISPR/Cas9) system, increased levels of HDL cholesterol were observed in the pigs. When fed a high-fat, high-cholesterol diet, the pigs developed significant hypercholesterolemia and progressive atherosclerotic lesions (Fang et al., 2018).

Another gene identified is *FABP1*, a member of the FABP family, which plays a direct role in the conversion of fatty acids into eicosanoid intermediates, as well as in the stabilization of leukotriene, and is also a biomarker of liver injury or liver-damaging stress (Furuhashi and Hotamisligil, 2008; Ishimura et al., 2013). In human studies, this gene showed positive results with blood pressure, triglycerides and AST (Ishimura et al., 2013), while in this study we showed co-expression in modules related to HDL. Studies in young adults agreed that low HDL cholesterol levels and hypertriglyceridemia were associated with serum *FABP1* levels, suggesting it as a possible circulating biomarker of adiposity and metabolic diseases associated with insulin resistance, involved in hepatic lipid binding and lipid metabolism (Shi et al., 2012). In the SOY1.5 group, the hub gene of the *Darkturquoise* module, *DCXR*, acts as an enzyme mediating the reductive metabolism of toxic reactive and toxic carbonyl compounds, already associated with diseases such as diabetes (Yang et al., 2017).

HDL plays a role in cytokine regulation, is part of the innate immune response and may have an atheroprotective effect by modulating the complement system (Yu et al., 2010). In addition to its antiatherogenic properties, it is associated with several immunomodulatory effects due to HDL ability to remove free cholesterol from the cell membrane. These findings are corroborated by the fact that the co-expressed hub gene is *IF144*, which is involved in the immune response. The main pathways enriched in this context were the immune system process and the positive regulation of interleukin-6 production. In people over 65 years of age, IL-6 is one of the factors that may influence the contribution of low HDL-C levels, as IL-6 has effects related to modifying the activity of triglyceride lipases (Zuliani et al., 2007). In relation to the SOY3.0 group, the correlation with this module was negative.

### 4.4 Key enrichment and hub gene related to globulins, cholesterol, AST and total proteins

We identified a negative correlation with globulins in the SOY1.5 group, along with enrichment in endocytosis, GTP binding, and translation initiation factor activity. In addition, we observed a module positively associated with cholesterol in the SOY1.5 group, with genes enriched in pathways such as cholesterol metabolism, and the *SYCP3* gene was the hub gene identified, where knockdown of this gene affects the expression of genes related to lipid metabolism (Manunza et al., 2014).

We identified AST and total protein as modules present exclusively in the SOY3.0 group, suggesting specific modulations in important biological processes in response to the oil level.

The *AKR1D1* hub gene, which has been identified in association with AST, is associated with steatosis and inflammation and regulates key metabolic processes. *In vitro* studies using human hepatoma cells have linked negative expression of *AKR1D1* to Nonalcoholic fatty liver disease (NAFLD) (Nikolaou et al., 2019).

Modulation of co-expression by diets with different oil content: By applying WGCNA and analyzing the RNA-Seq data, we can examine how FA were specifically modulated in each diet, improving our understanding of what happened in the differential expression analysis (Fanalli et al., 2022c), where we identified several of them enriched in interesting pathways.

Briefly, in the SOY1.5 group, we identified genes with positive co-expression with albumin that may play specific roles in biological processes related to immune response, regulation of intracellular signaling cascades, and control of gene expression. Highlighting the diversity of genetic interactions and biological processes affected by the higher concentration of soybean oil, we identified positive co-expression in SOY3.0 that may be involved in specific biological processes such as Alzheimer’s disease, immune response, RNA binding, NF-kappaB binding. These effects differ from those observed in SOY1.5, where gene expression varied between groups, possibly due to the inclusion of oil at different concentrations and may be related to the effects of FA composition in modulating the properties of the membrane as well as some of the membrane lipids.

It has also been reported that membrane lipids are important for cellular maintenance and that changes or disorders in these lipids may have consequences in the brain, as the liver serves as a central organ for systemic metabolism (De Carvalho and Caramujo, 2018; Fanalli et al., 2022c).

The difference in co-expression observed in relation to HDL in the SOY1.5 and SOY3.0 groups suggests a different regulatory pattern for HDL in response to the higher concentration of soybean oil.

The results obtained illustrate how FA can modulate gene expression and biological processes in response to different dietary compositions, revealing a complex relationship between diet, blood phenotypes, and molecular pathways in pigs. Our study clearly demonstrates that different dietary FA profiles induce changes in tissue-specific expression profiles to alter the complex network of co-expression. By highlighting the importance of FA in health, our findings underscore the practicality of using serum biochemical parameter sampling. Further studies are warranted to explore potential applications for optimizing swine production and disease management.

## 5 Conclusion

We used a weighted gene co-expression network to investigate the effects of different diets on expression profiles. This resulted in altered gene interaction and co-expression patterns within the transcriptome. Our study also identified key pathways related to cardiovascular and neurodegenerative diseases, as well as immune responses, metabolic pathways and cholesterol metabolism, which showed correlations with biochemical parameters of pig blood serum. We identified key genes co-expressed with albumin such as *CCL5, PNISR*, *ENSSCG00000047967*, and globulins such as *DEGS1*. We also observed genes co-expressed with HDL such as *DCXR* and *IF144.* These correlations were observed in the context of two different levels of soybean oil supplementation, specifically 1.5% and 3%. These findings provide valuable insights into the complex effects of dietary FA profile on gene expression and metabolic pathways in pigs and contribute to our understanding of genetic and metabolic responses to dietary variation. These findings are also crucial for understanding the genetic and metabolic responses to dietary modification and contribute to the development of more precise nutritional strategies.

## Data Availability

The original contributions presented in the study are included in the article/Supplementary Material, further inquiries can be directed to the corresponding author.
