# Supplementary material for: Key co-expressed genes correlated with blood serum parameters of pigs fed with different fatty acid profile diets
Source: Front Genet. 2024 Jul 3;15:1394971. doi: 10.3389/fgene.2024.1394971 (PMC11252010; doi:10.3389/fgene.2024.1394971)
Supplement: Supplementary file 3 [file Table1.DOCX]

**Table S1**: Composition of the experimental diets (as-fed basis).

|  | Grower I | |  | Grower II | |  | Finisher I | |  | Finisher II | |  | Finisher III | |  | Finisher IV | |
| --- | --- | --- | --- | --- | --- | --- | --- | --- | --- | --- | --- | --- | --- | --- | --- | --- | --- |
|  | (day 0 to 21) | |  | (day 21 to 42) | |  | (day 42 to 56) | |  | (day 56 to 63) | |  | (day 63 to 70) | |  | (day 70 to 98) | |
| Item | SOY1.5 | SOY3.0 |  | SOY1.5 | SOY3.0 |  | SOY1.5 | SOY3.0 |  | SOY1.5 | SOY3.0 |  | SOY1.5 | SOY3.0 |  | SOY1.5 | SOY3.0 |
| Ingredient, % |  |  |  |  |  |  |  |  |  |  |  |  |  |  |  |  |  |
| Corn, 7.5% CP^1^ | 63.47 | 61.88 |  | 66.40 | 64.71 |  | 69.13 | 67.54 |  | 69.63 | 68.04 |  | 69.59 | 68.00 |  | 70.09 | 68.50 |
| Soybean meal, 46% CP | 28.33 | 28.42 |  | 26.10 | 26.29 |  | 23.37 | 23.46 |  | 23.37 | 23.46 |  | 22.93 | 23.02 |  | 22.93 | 23.02 |
| Meat and bone meal, 44% CP | 3.00 | 3.00 |  | 3.00 | 3.00 |  | 3.00 | 3.00 |  | 3.00 | 3.00 |  | 3.00 | 3.00 |  | 3.00 | 3.00 |
| Fat source | 1.50 | 3.00 |  | 1.50 | 3.00 |  | 1.50 | 3.00 |  | 1.50 | 3.00 |  | 1.50 | 3.00 |  | 1.50 | 3.00 |
| Dicalcium phosphate | 0.55 | 0.56 |  | 0.56 | 0.57 |  | 0.26 | 0.27 |  | 0.26 | 0.27 |  | 0.27 | 0.27 |  | 0.26 | 0.27 |
| Limestone | 0.43 | 0.42 |  | 0.38 | 0.38 |  | 0.84 | 0.84 |  | 0.76 | 0.75 |  | 0.84 | 0.84 |  | 0.69 | 0.69 |
| Salt | 0.50 | 0.50 |  | 0.50 | 0.50 |  | 0.50 | 0.50 |  | 0.50 | 0.50 |  | 0.50 | 0.50 |  | 0.50 | 0.50 |
| Vitamin-mineral premix^2^ | 1.61 | 1.61 |  | 1.08 | 1.08 |  | 1.01 | 1.01 |  | 0.60 | 0.60 |  | 1.02 | 1.01 |  | 0.66 | 0.65 |
| L-Lysine.HCl | 0.35 | 0.35 |  | 0.29 | 0.29 |  | 0.25 | 0.25 |  | 0.25 | 0.25 |  | 0.20 | 0.20 |  | 0.20 | 0.20 |
| DL-Methionine | 0.11 | 0.11 |  | 0.07 | 0.08 |  | 0.04 | 0.04 |  | 0.04 | 0.04 |  | 0.02 | 0.02 |  | 0.03 | 0.03 |
| L-Threonine | 0.14 | 0.15 |  | 0.11 | 0.11 |  | 0.09 | 0.09 |  | 0.09 | 0.09 |  | 0.06 | 0.06 |  | 0.06 | 0.07 |
| L-Tryptophan | 0.01 | 0.01 |  | - | - |  | - | - |  | - | - |  | - | - |  | - | - |
|  |  |  |  |  |  |  |  |  |  |  |  |  |  |  |  |  |  |
| Ractopamine.HCl, 2% | - | - |  | - | - |  | - | - |  | - | - |  | 0.08 | 0.08 |  | 0.08 | 0.08 |
| Calculated composition^3^ |  |  |  |  |  |  |  |  |  |  |  |  |  |  |  |  |  |
| Metabolizable energy, Mcal/kg | 3.28 | 3.36 |  | 3.29 | 3.36 |  | 3.28 | 3.36 |  | 3.29 | 3.36 |  | 3.28 | 3.35 |  | 3.29 | 3.36 |
| SID^4^ Lysine, % | 1.15 | 1.15 |  | 1.05 | 1.05 |  | 0.95 | 0.95 |  | 0.95 | 0.95 |  | 0.90 | 0.90 |  | 0.90 | 0.90 |
| SID Methionine + Cysteine, % | 0.62 | 0.62 |  | 0.57 | 0.57 |  | 0.51 | 0.51 |  | 0.51 | 0.51 |  | 0.49 | 0.49 |  | 0.49 | 0.49 |
| SID Threonine, % | 0.75 | 0.75 |  | 0.68 | 0.68 |  | 0.63 | 0.63 |  | 0.63 | 0.63 |  | 0.58 | 0.59 |  | 0.59 | 0.59 |
| SID Tryptophan, % | 0.22 | 0.22 |  | 0.20 | 0.20 |  | 0.18 | 0.18 |  | 0.18 | 0.18 |  | 0.18 | 0.18 |  | 0.18 | 0.18 |
| Calcium, % | 0.84 | 0.84 |  | 0.81 | 0.81 |  | 0.80 | 0.80 |  | 0.77 | 0.77 |  | 0.80 | 0.80 |  | 0.75 | 0.75 |
| Available Phosphorous, % | 0.42 | 0.42 |  | 0.42 | 0.42 |  | 0.34 | 0.34 |  | 0.34 | 0.34 |  | 0.34 | 0.34 |  | 0.34 | 0.34 |
| Analyzed composition, % |  |  |  |  |  |  |  |  |  |  |  |  |  |  |  |  |  |
| CP | 19.71 | 19.64 |  | 18.75 | 18.71 |  | 17.66 | 17.58 |  | 17.66 | 17.59 |  | 17.41 | 17.33 |  | 17.42 | 17.35 |
| Ether extract | 4.49 | 5.49 |  | 4.03 | 5.53 |  | 3.63 | 6.57 |  | 3.63 | 5.59 |  | 3.16 | 5.61 |  | 3.16 | 5.61 |

^1^CP = crude protein.

^2^Provided per kilogram of diet: 6.500 UI vitamin A; 1.800 UI vitamin D_3_; 30 UI vitamin E; 2 mg vitamin K_3_; 1.2 mg vitamin B_1_; 3.4 mg vitamin B_2_; 2.0 mg vitamin B_6_; 125 mg Cu; 80 mg Fe; 40 mg Mn; 0.35 mg Se; 1.25 mg Zn.

^3^Calculated according to Rostagno et al. (2011).

^4^SID = standardized ileal digestible. Adapted from Almeida et al., 2021
